# The Nature of the Hymen

**Published:** 1888-02

**Authors:** Bland Sutton

**Affiliations:** London, Eng.


### *THE NATURE OF THE HYMEN.*

By Dr. BLAND SUTTON, of London, Eng.

In remarks before a recent meeting of the British Gynecological Society, the author pointed out that the hymen was formed in the same way as the rectum, that is, by the invagination of the epiplastic layer which comes into contact with, and fuses with, the primitive gut in its posterior segment. The distinction between the two remains throughout life, for, in the rectum, a ridge of adenoid tissue marks the situation where the squamous epithelium of the angle joins the columner cells of the rectum. In the vagina, the corresponding spot was indicated by the hymen or its remains. The mode by which the proctodoceum and the gut become fused, is as follows: When the two culs-de-sac come in contact and exercise pressure on each other, the edges gradually cohere and join organically. At this stage, the lumen is still obstructed with a thin septum ; this gradually became thinner from pressure, and, ultimately, perforation took place. The hole increased and the septum slowly disappeared until a complete channel was the result.

In the case of the anus, it was clear that if the invagination failed to reach the gut, an imperforate rectum would be the result. From the study of those conditions, it was clear that the hymen was merely a thin septum resulting from the imperfect coalescence of the proctodoceum with the uro-genital section of the cloaca. Should the septum be complete, it would be termed an imperforate hymen. Occasionally, the perforation was multiple, concentric or cribriform.

Similar evidence as to the nature of the hymen was obtained from the opposite end of the alimentary canal. The mouth and pharynx, with the associated structures, were derived from an involution of the surface epiblast, named the stomodicum. This met the blind anterior end of the foregut at a spot eventually coinciding with the cricoid cartilage. Should those parts fail to unite, an imperforate pharynx was the result. When the coalescence was imperfect, then a hymen-like diaphragm might be found. So far as the alimentary canal was concerned, diaphramata of this nature occasionally existed between the pharynx and the esophagus in the duodenum, immediately above the entrance of the bile duct, and in the rectum at the point of union of the anus and gut.

Thus the formation of a diaphragm, sometimes complete, but more commonly perforated at the entrance of the vagina, was only in agreement with what occurred in other parts of the body, when two culs-de-sac coalesced to form a continuous passage. Lastly, abnormalities of the vaginal orifice were not infrequently associated with defects of the alimentary canal. This might be expected, taking into consideration the embryological relation of the part with the cloaca.

